# Potential Impact of Statins on Neuronal Stress Responses in Patients at Risk for Cardiovascular Disease

**DOI:** 10.3390/jpm11040261

**Published:** 2021-04-01

**Authors:** Flavia Diggelmann, Susan Bengs, Achi Haider, Gioia Epprecht, Anna Luisa Beeler, Dominik Etter, Winandus J. Wijnen, Angela Portmann, Geoffrey I. Warnock, Valerie Treyer, Muriel Grämer, Atanas Todorov, Nidaa Mikail, Alexia Rossi, Tobias A. Fuchs, Aju P. Pazhenkottil, Ronny R. Buechel, Felix C. Tanner, Philipp A. Kaufmann, Catherine Gebhard, Michael Fiechter

**Affiliations:** 1 Department of Nuclear Medicine, University Hospital Zurich, 8091 Zurich, Switzerland; flavia.diggelmann@usz.ch (F.D.); susan.bengs@usz.ch (S.B.); ahmed.haider@usz.ch (A.H.); gioia.epprecht@usz.ch (G.E.); annaluisa.beeler@usz.ch (A.L.B.); dominik.etter@usz.ch (D.E.); winandus.wijnen@usz.ch (W.J.W.); angela.portmann@usz.ch (A.P.); geoffreyiain.warnock@usz.ch (G.I.W.); valerie.treyer@usz.ch (V.T.); muriel.graemer@usz.ch (M.G.); atanas.todorov@usz.ch (A.T.); nidaa.mikail@usz.ch (N.M.); alexia.rossi@usz.ch (A.R.); tobias.fuchs@ksa.ch (T.A.F.); aju.pazhenkottil@usz.ch (A.P.P.); ronny.buechel@usz.ch (R.R.B.); pak@usz.ch (P.A.K.); catherine.gebhard@usz.ch (C.G.); 2 Center for Molecular Cardiology, University of Zurich, 8952 Schlieren, Switzerland; felix.tanner@usz.ch; 3 Department of Cardiology, University of Zurich, 8091 Zurich, Switzerland; 4 Swiss Paraplegic Center, 6207 Nottwil, Switzerland

**Keywords:** amygdala, statins, inflammation, dyslipidaemia, PET

## Abstract

Background: Recent studies indicate that enhanced neuronal stress responses are associated with adverse cardiovascular outcomes. A chronic inflammatory state seems to mediate this detrimental neuro-cardiac communication. Statins are among the most widely prescribed medications in primary and secondary cardiovascular disease (CVD) prevention and not only lower lipid levels but also exhibit strong anti-inflammatory and neuroprotective effects. We therefore sought to investigate the influence of statins on neuronal stress responses in a patient cohort at risk for CVD. Methods: 563 patients (61.5 ± 14.0 years) who underwent echocardiography and ^18^F-fluorodeoxyglucose (^18^F-FDG) positron emission tomography (PET) were retrospectively identified. Metabolic activity of the amygdala, a part of the brain’s salience network, was quantified by ^18^F-FDG uptake, while normal cardiac morphology and function were assured by echocardiography. Vertebral bone marrow metabolism, a marker of inflammatory activity, was measured by ^18^F-FDG PET. Results: Increased neuronal stress responses were associated with an increased inflammatory activity in the bone marrow (r = 0.152, *p* = 0.015) as well as with a subclinical reduction in left ventricular ejection fraction (LVEF, r = −0.138, *p* = 0.025). In a fully-adjusted linear regression model, statin treatment was identified as an independent, negative predictor of amygdalar metabolic activity (B-coefficient −0.171, *p* = 0.043). Conclusions: Our hypothesis-generating investigation suggests a potential link between the anti-inflammatory actions of statins and reduced neuronal stress responses which could lead to improved cardiovascular outcomes. The latter warrants further studies in a larger and prospective population.

## 1. Introduction

Inflammation plays a fundamental role in the evolution and progression of cardiovascular disease [1,2]. Beyond their lipid-lowering properties, statins exert strong anti-inflammatory effects through inhibition of the mevalonate pathway [3]. Moreover, statins exhibit neuroprotective features by reducing neuroinflammatory responses such as suppression of nicotinamide adenine dinucleotide phosphate (NADPH) oxidase activity and decrease of pro-inflammatory cytokines. They further promote neurogenesis by modulation of Ras homolog family member A (RhoA), protein kinase B (Akt), and wingless-related integration site (Wnt) signalling pathways [4].

Complex neural functions such as cognition and emotion are influenced by a group of interconnected neural structures called the brain’s salience network. Particularly in emotional stress generation, this network is known to play an important role [5]. Activation of the brain’s salience network, which includes the amygdala as a key component [6], results in autonomic, hormonal, and behavioral changes, ulimately leading to anxiety and stress [7]. The efferent projections of the amygdala to the brainstem are involved in sympathetic responses to psychological stress [8]. Both an upregulation of bone marrow activity and inflammation of the arteries was proposed as a potential mechanism interconnecting increased amygdalar metabolism with an elevated risk of adverse cardiovascular events [7,9]. Since heightened resting activity of the amygdalais an independent predictor of cardiovascular outcome [7], we sought to investigate the effect of statin therapy on amygdalar metabolism and inflammation in an aged study cohort at risk for cardiovascular disease (CVD).

## 2. Material and Methods

**Figure 1:**
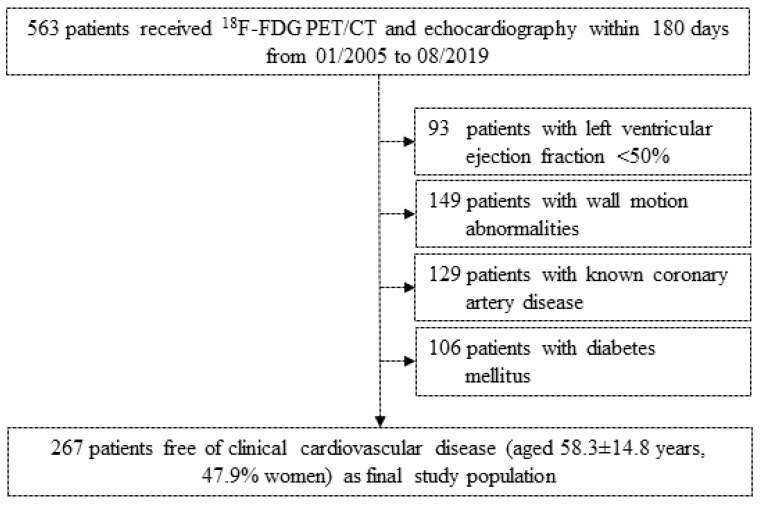
Clinical study cohort. Study flow chart visualizing initial complete study cohort and excluded patients on pre-defined criteria (left ventricular ejection fraction, motion abnormalities, known coronary artery disease, and diabetes mellitus). Totally 267 patients free of clinical cardiac disease entered the final study population for statistical investigation.

A total of 563 patients (aged 61.5 ± 14.0 years, 36.2% women) underwent both standardized echocardiography and full-body ^18^F-fluorodeoxyglucose (FDG) positron emission tomography (PET) / computed tomography (CT) to assess cardiac function and resting amygdalar glucose metabolism (mean time interval [±standard error, SE] 43.4 ± 2.0 days) [10]. After exclusion of 296 patients due to either the presence of chronic inflammatory disorders or active malignancies, impaired left ventricular ejection fraction (LVEF) <50% (*n* = 93), presence of wall motion abnormalities (*n* = 149), and/or known coronary artery disease (*n* = 129), and diabetes (*n* = 106), a cohort of 267 patients free of clinical CVD (aged 58.3 ± 14.8 years, 47.9% women) were retrospectively analyzed (Figure 1). Serial regions of interest (ROI) to measure ^18^F-FDG standardized uptake value (SUV) were placed around both amygdalae using the brain maximum probability map (“Hammersmith atlas” [11]), as previously described [12]. Normalization was then performed against cerebellar activity, which was determined by serial placement of ROI encompassing both the left and the right cerebellum. ^18^F-FDG bone marrow was retrieved by serial placement of standardized ROI (10 mm diameter) in the center from the first thoracic to the fifth lumbar vertebral body. Image analysis was performed by using PMOD software V4.1 (PMOD Technologies LLC, Zurich, Switzerland). Statistical analysis was performed using IBM SPSS, version 25.0 (IBM Corp, Armonk, NY, USA). Pearson product-moment test was applied to identify associations, and after confirming normal distribution of data and excluding collinearity issues, stepwise linear regression models were used to identify predictor variables for amygdalar ^18^F-FDG uptake (smallest probability of F ≤ 0.05, largest probability of F ≥ 0.1). As this is an explorative investigation, no pre-specified level of significance was applied. Thus, the level of evidence was quantified continuously as recommended [13].

## 3. Results

No significant differences in baseline characteristics were observed between patients with and without statin therapy, except for age (*p* < 0.001) and dyslipidaemia (*p* = 0.003, Table 1). The average statin dose taken was 28.8 ± 18.0 mg in our cohort.

**Figure 2:**
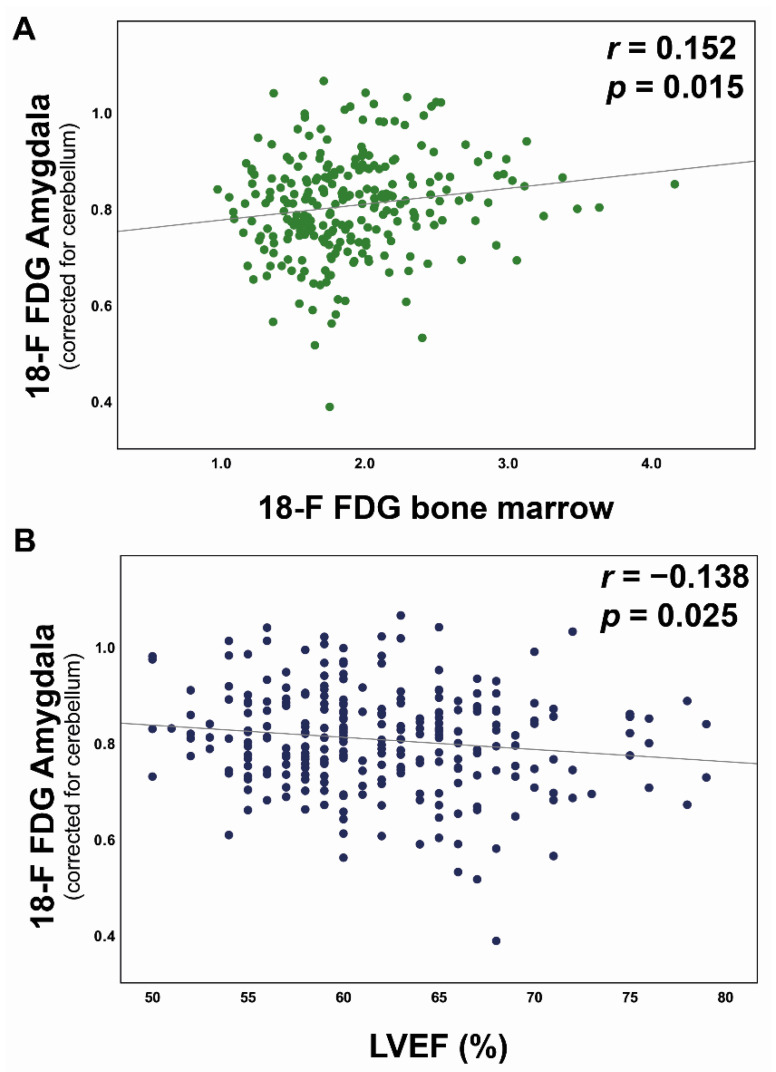
Association of resting amygdala activity with bone marrow metabolism and cardiac function. Pearson product-moment correlation of ^18^F-FDG amygdala uptake (standardized uptake value ratio, SUVR) shows a positive correlation with bone marrow activity (standardized uptake value, SUV, (**A**)) and a negative association with left ventricular ejection fraction (LVEF, %, (**B**)). Correlation coefficients and *p*-values are indicated. ^18^F-FDG, ^18^F-fluorodeoxyglucose.

As previously demonstrated [14], an enhanced bone marrow ^18^F-FDG uptake was associated with increased plasma C-reactive protein (CRP) levels (r = 0.384, *p* < 0.001) supporting the notion that augmented bone marrow metabolic activity indicates an inflammatory state. Further, a positive association of resting amygdalar activity with vertebral bone marrow ^18^F-FDG uptake (r = 0.152, *p* = 0.015, Figure 2A) and a negative correlation between amygdalar uptake and LVEF (r = −0.138, *p* = 0.025, Figure 2B) were detected. In contrast to patients without statin therapy, these correlations were no longer evident in patients on statin therapy (*n* = 42, *p* = not significant).

In a first stepwise linear regression model adjusted for CVD risk factors including dyslipidaemia (*n* = 18), hypertension (*n* = 82), obesity (*n* = 39), positive family history for cardiovascular disease (*n* = 3), and smoking (*n* = 41), both enhanced ^18^F-FDG bone marrow uptake and reduced LVEF were selected as predictors of an increased amygdalar activity (B-coefficient for bone marrow uptake: 0.150, *p* = 0.016; for LVEF: −0.127, *p* = 0.041). In a second stepwise linear regression model that included the predictor variables age, CVD risk factors, medications (angiotensin converting enzyme inhibitors [*n* = 38], angiotensin II receptor blockers [*n* = 38], beta blockers [*n* = 65], loop diuretics [*n* = 41], aspirin [*n* = 52], and statins [*n* = 42]), as well as kidney function, statins remained in the model as the only independent predictor of amygdalar activity (B-coefficient: −0.171, *p* = 0.043).

**Figure 3:**
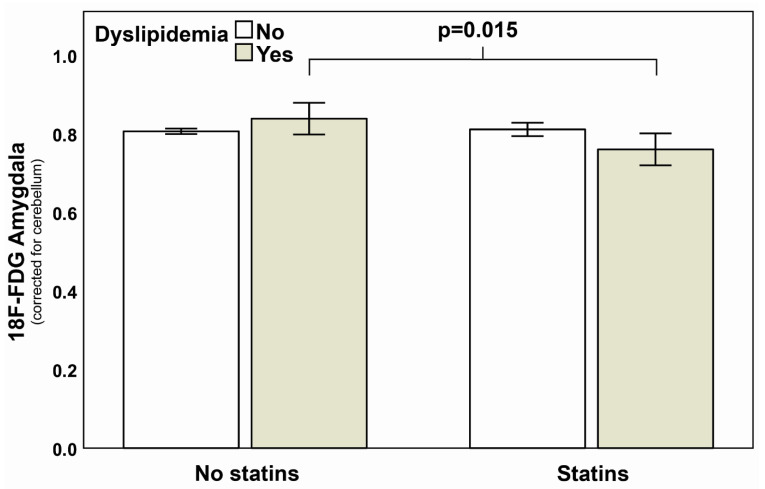
Impact of statins on amygdalar activation pattern. In a three-way ANOVA, an interaction term consisting of statins and dyslipidaemia was associated with a blunted amygdalar activity (*p* = 0.015). Interaction terms consisting of sex, statins and/or dyslipidaemia were statistically not evident (*p* > 0.05). The first left two bars show resting amygdalar activity of patients without statins and the right two bars depict patients with statins therapy. Further, white bars correspond to those patients without and grey bars to those patients with dyslipidemia. For reasons of improved readability, the variable “sex” was not included in this Figure. Error bars display standard error, SE. *p*-value is depicted.

In a three-way ANOVA analysis with ^18^F-FDG amygdalar metabolism as dependent and statins, dyslipidaemia, and sex as independent variables, both statin (*p* = 0.024) and sex (*p* = 0.038) led to significantly different metabolic activity of the amygdala, whereas dyslipidaemia alone (*p* = 0.584) and the combination of all three variables (*p* = 0.193) had no influence on amygdalar metabolism. However, the presence of statins in combination with dyslipidaemia was associated with the most distinct difference in amygdalar metabolism resulting in a lower mean amygdalar activity as compared to the absence of statins and dyslipidaemia (0.76 ± 0.12 vs. 0.81 ± 0.10, *p* = 0.015, Figure 3).

## 4. Discussion

We demonstrate that statin therapy is associated with reduced amygdalar activity in aged patients at risk for CVD. We further confirm previous findings [7] by reporting an association of amygdalar activity with inflammatory processes via an enhanced production of inflammatory progenitor cells in the bone marrow and the relation of vertebral bone marrow metabolism with plasma CRP levels. Moreover, the association between an elevated metabolic activity of the amygdala and a subclinical reduction in LVEF in our study suggests a detrimental link between an enhanced neuronal stress response and impaired cardiac function. The latter is in line with the observation that persistent activation of β-adrenergic receptors leads to an upregulation of cytokines, macrophage infiltration, and subsequent cardiac fibrosis by inflammasome-dependent activation of interleukin-18 in myocardial cells [15]. Notably, statins inhibit the 3-hydroxy-3-methyl-glutaryl-coenzyme A (HMG-CoA) reductase, the rate-limiting enzyme in the mevalonate pathway. Downstream metabolites of this pathway are essential for the attachment of guanosine triphosphatases (i.e., rat sarcoma [Ras], Rac, and ras homolog family member A [RhoA]) to the cell membrane and play critical roles in various steps of immune cell activation, metabolism, and survival [16]. Among individuals who received statin therapy, resting amygdalar activity was no longer associated with ^18^F-FDG bone marrow uptake. Conversely, among those who did not receive statin therapy, the association of enhanced resting amygdalar activity with higher ^18^F-FDG bone marrow uptake and reduced LVEF was retained. These observations suggest that the deleterious effects of the neuro-hematopoetic axis on the cardiovascular system might be modifiable by optimum medical therapy. Proprotein convertase subtilisin/kexin type 9 (PCSK9), an enzyme involved in low-density lipoprotein receptor degradation has been shown to not only promote early atherosclerosis but also being ubiquitously present in the body including neural tissue [17].

Given that selective inhibition of PCSK9 by monoclonal anti-bodies effectively reduced levels of cholesterol and adverse cardiovascular events, the involvement of PCSK9 in lipid metabolism in conjunction with the downregulating effects of statins on amygdalar metabolism merits further investigation.

Several limitations of our study need to be pointed out: First, this is a single-center and retrospective cohort study with a purely observational design and thus carries a limited generalizability of its results as well as a potential for referral bias. Second, patients did not receive magnetic resonance imaging of neural structures. Thus, limitations apply for regional mapping of the amygdala. Further, partial volume correction was not conducted. Third, age-based neurodegeneration and brain atrophy may act as confounders in this study. Fourth, although patients with impaired LVEF or clinical cardiovascular disease were excluded from our study, a slightly elevated overall N-terminal pro-B-type natriuretic peptide (NT-proBNP) level was observed in our cohort. Increased NT-proBNP concentrations are present in many non-cardiac conditions such as pulmonary or renal disease and are commonly seen in older patients. Therefore, given the increased age and the higher prevalence of comorbidities and CVD risk factors in our study cohort, the possibility that unmeasured variables have influenced our findings cannot be completely ruled out. Fifth, the exact duration of statin therapy was not reliably retrievable from our data sets. Thus, no conclusions can be made concerning the duration of statin therapy and its effect dynamics on amygdalar activity. Therefore, the findings of our investigation should be regarded as hypothesis-generating and will have to be further investigated in a larger and ideally prospective cohort trial.

## 5. Conclusions

Given the anti-inflammatory properties of statins, our observation of a reduced neuronal stress response in patients on statin therapy suggests a novel mechanistic link on how statins could exert cholesterol-independent, “pleiotropic” effects on cardiovascular health. The modulation of neuronal stress responses by statins and the potential cardioprotective effect of this association provides new insights into the highly investigated neuro-haematopoietic-vascular axis and might help to explore new concepts of primary and secondary CVD prevention.

## Figures and Tables

**Table 1:** Patient baseline characteristics.

Patient Baseline Characteristics	Total (*n* = 267)	Statin (*n* = 42)	No Statin (*n* = 225)	*p*-Value (Statin vs. No Statin)
Age (years), mean ± SD	58.3 ± 14.8	66.1 ± 10.2	56.9 ± 15.1	<0.001
Hypertension, *n*(%)	82(30.7)	16(6.0)	66(24.7)	0.227
Dyslipidemia, *n*(%)	18(6.7)	8(3.0)	10(3.7)	0.003
Obesity, *n*(%)	39(14.6)	6(2.2)	33(12.4)	1.000
Family history of CAD, *n*(%)	3(1.1)	1(0.4)	2(0.7)	0.403
Smoking, *n*(%)	41(15.4)	7(2.6)	34(12.7)	0.816
Alcohol, *n*(%)	18(6.7)	1(0.4)	17(6.4)	0.323
LVEF (%), mean ±SD	61.8 ± 5.8	61.6 ± 5.5	61.8 ± 5.9	0.829
Blood glucose (mmol/L), mean ± SD	5.6 ± 1.3	5.4 ± 0.9	5.7 ± 1.4	0.272
Creatinine (µmol/L), mean ± SD	102.3 ± 90.4	118.1 ± 123.7	99.6 ± 83.6	0.387
CRP (mg/L), mean ± SD	53.1 ± 68.9	63.2 ± 82.1	51.1 ± 66.4	0.454
NT-proBNP (ng/L), mean ± SD	1553 ± 2468	2062 ± 3383	1468 ± 2367	0.664
Neutrophiles (×10^3^/µL), mean ± SD	5.85 ± 3.51	6.62 ± 3.41	5.72 ± 3.53	0.300
Lymphocytes (×10^3^/µL), mean ± SD	1.34 ± 0.74	1.32 ± 0.66	1.34 ± 0.75	0.900
^18^F-FDG bone marrow (SUV), mean ± SD	1.91 ± 0.50	1.85 ± 0.43	1.92 ± 0.51	0.424
^18^F-FDG amygdala (SUV), mean ± SD	0.81 ± 0.10	0.80 ± 0.10	0.81 ± 0.10	0.722

CAD, coronary artery disease; CRP, C-reactive protein; ^18^F-FDG, ^18^F-fluorodeoxyglucose; LVEF, left ventricular ejection fraction; NT-proBNP, N-terminal pro-B-type natriuretic peptide; SD, standard deviation; SUV, standardized uptake value. Data are displayed as mean ± SD or frequencies (in percentage). Two-sided *p*-values are depicted. Percentage values refer to the total patient number of 267.

## Data Availability

All relevant data are within the manuscript and its supporting information files.
